# Statistical learning of phonotactics by children can be affected by another statistical learning task

**DOI:** 10.1017/s0142716423000449

**Published:** 2023-11-28

**Authors:** Peter T. Richtsmeier, Lisa Goffman

**Affiliations:** 1 Communication Sciences and Disorders, Oklahoma State University, Stillwater, OK, USA; 2 Callier Center for Communication Disorders, Behavioral and Brain Sciences, University of Texas at Dallas, Dallas, TX, USA

**Keywords:** child speech development, experiment interaction, phonology, phonotactics, statistical learning

## Abstract

Children typically produce high-frequency phonotactic sequences, such as the /st/ in “toaster,” more accurately than the lower frequency /mk/ in “tomcat.” This high-frequency advantage can be simulated experimentally with a statistical learning paradigm, and when 4-year-old children are familiarized with many examples of a sequence like /mk/, they generally produce it more accurately than if they are exposed to just a few examples. Here, we sought to expand our understanding of the high-frequency advantage, but surprisingly, we instead uncovered an exception. Twenty-nine children between 4 and 5 years of age completed a phonotactic statistical learning experiment, but they also completed a separate experiment focused on statistical learning of prosodic contours. The order of the experiments was randomized, with the phonotactic statistical learning experiment occurring first for half of the children. For the children who completed the phonotactic learning experiment first, the results were consistent with previous research and a high-frequency advantage. However, children who completed the phonotactic learning experiment second produced low-frequency sequences more accurately than high-frequency sequences. There is little precedent for the latter effect, but studies of multistream statistical learning may provide some context for unpacking and extending the result.

## Introduction

An important component of language development in the preschool years is achieving adult levels of speech production accuracy. Children who are acquiring a broad range of languages achieve near-adult accuracy by the age of 5 years (McLeod & Crowe, 2018), with smaller increases in accuracy (Smit, 1993; Smit et al., 1990) and intelligibility (Hustad et al., 2021) occurring through age 10 years.

A number of factors appear to guide the development of speech accuracy, including speech motor development (Namasivayam et al., 2020; Smith & Zelaznik, 2004; Stokes & Surendran, 2005), perceptual learning (Ota & Green, 2013), genetic factors (for relevant discussion see Terband et al., 2019, p. 218), functional load (Van Severen et al., 2013), and language-wide frequency (Edwards & Beckman, 2008). Regarding the final factor, children tend to have greater accuracy when producing sounds and sound sequences that are frequent in the ambient language. For example, Munson (2001) had 3-year-olds, 8-year-olds, and adults produce nonwords with a CVCCVC shape. The word-medial consonant sequences were controlled for their frequency in English, with sequences like /st/ occurring frequently and sequences like /mk/ occurring infrequently. Across age groups, production accuracy exhibited a *high-frequency advantage*. That is, children and adults were more accurate to produce high-frequency sequences, and they also produced them faster and with less variability. The high-frequency advantage has been reported in children with speech sound disorders (Munson et al., 2005), in a variety of other languages (Edwards & Beckman, 2008; Masdottir & Stokes, 2016), and is consistent with a broader literature showing frequency effects in language acquisition (Ambridge et al., 2015).

The high-frequency advantage has also been simulated experimentally in the laboratory in a statistical learning paradigm with young children. Richtsmeier et al. (2011) used a familiarization-then-test paradigm that is common in infant statistical learning studies. However, they measured phonotactic learning by familiarizing children with one set of words and looking at production accuracy for a target word-medial consonant sequence in novel test words. Frequency in the familiarization was controlled, and “high frequency” was operationalized in three different ways across three different experiments: the first experiment focused on high-token frequency with talker variability or lots of talkers saying /nʌmkət/. The second experiment combined word-type frequency with talker variability or several talkers each saying /dɪmkəs/, /gumkən/, and /nʌmkət/. The third experiment examined word-type frequency without talker variability or one talker per word saying /dɪmkəs/, /gumkən/, and /nʌmkət/. Children were more accurate to produce word-medial sequences like /mk/ in the novel test word /sæmkəf/ that they heard in multiple familiarization sequences produced by multiple talkers in the second experiment. There were no equivalent learning effects in the high-token frequency first experiment or in the word-type-only third experiment. Thus, phonologically unique words combined with talker variability appear able to mirror language-wide frequency effects.

To summarize the review up to this point, a high-frequency advantage is observed when children more accurately produce sounds and sound sequences that are frequent in a language compared with those that are infrequent. Furthermore, an experimental high-frequency advantage has been observed by manipulating the frequency of sound sequences within a statistical learning experiment. These findings reveal sensitivity to statistical characteristics of the input following naturalistic exposure, as well as following a brief exposure session in a laboratory task. Researchers argue that the short-term sensitivity feeds into long-term sensitivity (Pierrehumbert, 2003). Furthermore, these sensitivities may shift over the course of development and have broader impacts including the facilitation of lexical and grammatical learning (Breen et al., 2019; Gerken et al., 2019; Gómez & Gerken, 2000).

The present study was intended to extend knowledge of the high-frequency advantage in two ways. First, if short-term statistical learning contributes to the long-term advantages for frequent sequences of a language observed by Munson (2001) and others, the short-term statistical learning effects should persist over time. Recently, Gómez (2017) has argued that retention of statistical learning is relatively understudied and deserves greater attention. In their daily lives, infants and children are exposed to a sea of statistical information. How the structure present in that voluminous input is learned and retained is of central importance to understanding how children learn their native language. Thus, one goal of this study was to examine statistical learning effects across multiple days, in this case, in two sessions 1 week apart. The second goal was to examine learning of statistical patterns other than phonotactic probabilities, in this case, prosodic contours. Goffman and colleagues have shown that children lack adult levels of sophistication when producing stress modulations for trochees like the noun form of *record* (REC.ord) as well as iambs like the verb form (re.CORD; Goffman, 1999; Goffman et al., 2007; Goffman & Malin, 1999). Therefore, the same children participating in the current study on phonotactic learning also engaged in a second study reported elsewhere, that was concerned with an input focusing on prosodic contours. Importantly, the order of the experiments was counterbalanced, with the phonotactic learning experiment occurring first for half of the children.

Extending an established high-frequency advantage, by examining it over time, was the intended purpose of this study. To foreshadow our results, a surprising and unanticipated result emerged. As expected, we found some evidence for a high-frequency advantage, but it was only observed in the participants who completed the phonotactic learning experiment first. For the participants who completed the phonotactic learning experiment second, there appeared to be a *low-frequency advantage* or, alternatively, a *high-frequency disadvantage*. Those participants were less accurate to produce sequences that they heard more often during the familiarization relative to the sequences they heard just once. In the general discussion, we take up why participants might more accurately produce sequences that they have heard less often, why the order of the two experiments might be relevant, and how the results might correspond to a small but growing literature on multistream statistical learning, or learning paradigms that involve competing inputs (e.g., Weiss et al., 2009). We will argue that, as in the real world, it is important to extend statistical learning studies to incorporate multiple, and potentially competing, cues in the input.

## Method

### Participants

A total of 41 children between the ages of 4 and 5 years were recruited for the study. Children under six continue to produce speech errors, especially in novel and complex words, and they are still developing their knowledge of phonology (McLeod & Crowe, 2018). Therefore, this age group was ideal for measuring learning via changes to speech accuracy. Ten children were not included in the analyses because they did not participate for all 5 days, and they left one or both experiments incomplete. Two additional children were removed because standardized testing indicated that they had a speech sound disorder. The remaining 29 children (17 females and 12 males) were included in the analyses. Summary data appear in Table 1.

All participants met the following criteria for typical development. All children passed a hearing screening of pure tones at 500, 1,000, 2,000, and 4,000 Hz at 20 dB. All children received standardized test scores at or above one standard deviation below the mean (standard scores ≥ 85). Additionally, parents were asked about their child’s development, and for the 29 participants included, no concerns were raised.

Standardized assessment and normative data were collected across a range of areas: speech production (Bankson-Bernthal Test of Phonology, Bankson & Bernthal, 1990), nonverbal cognition (Columbia Mental Maturity Scale; CMMS; Burgemeister et al., 1972), receptive vocabulary (Peabody Picture Vocabulary Test-4; PPVT-4; Dunn & Dunn, 2007), expressive vocabulary (Expressive Vocabulary Test; EVT; Williams, 2007), expressive syntax (Structured Photographic Expressive Language Test-3; SPELT-3; Dawson et al., 2003), and nonword repetition accuracy (Dollaghan & Campbell, 1998). Table 1 provides these data and the participants’ mean age in months and their age range. Average accuracy on the phonotactic experimental task is also included. Because a critical variable in this study is the order in which children completed this experiment relative to another experiment, the normative and experimental data are presented in separate columns for the phonotactics first and phonotactics second groups, and a *t*-test comparison between the groups is presented in the rightmost column. No significant differences, operationalized as *p* < .05, were observed for the normative data, but a χ2 comparison of the sex ratios indicated that there was an unplanned preponderance of females completing the phonotactic learning experiment first. We return to this imbalance in the results and general discussion. We also note that scores from the CMMS, the PPVT-4, the EVT, and the SPELT-3 indicate that this group of participants as a whole possessed above-average cognitive and language skills.

### Materials

This study relies on speech production to measure learning, and the target patterns that children produced were word-medial consonant sequences (Munson, 2001). The familiarization and test materials for the phonotactic learning experiment are presented in Table 2. Readers interested in the materials, procedures, and results from the prosodic learning experiment are encouraged to review them in the following preprint: https://doi.org/10.20944/preprints202105.0777.v1. To briefly summarize, in the prosody experiment the statistical pattern children were exposed to included two different stress patterns, one strong-weak as in /po.fə/ and the second weak-strong as in /bə.mi/. The full set of materials from the prosodic learning experiment appears in Appendix A. What is most relevant in the present context is that in the prosody experiment, overarching suprasegmental, or stress, statistical cues were relevant. In the phonotactic learning experiment that is the focus of the present work, phonotactic, or segmental, statistical cues were relevant.

The target consonant sequences appeared in nonsense words (hereafter referred to as “items”) with a CVCCVC shape and stress on the first syllable. All items started with a unique CV sequence and differed from other items by at least three phonemes. Both familiarization and test items were paired with colorful make-believe animals (Ohala, 1999), and children were told that the items were the names of the animals. Consonant sequences were chosen because they are relatively difficult to produce, making it likely that children would sometimes produce them in error, and learning could be measured. In addition, phonotactic frequency can readily be manipulated or controlled in CC sequences. Note that the phonotactics first data were reported by the authors in their 2017 publication. Data from the phonotactics second condition have not been reported elsewhere.

The phone and biphone frequencies for items were calculated using the online Phonotactic Probability Calculator (Vitevitch & Luce, 2004), https://calculator.ku.edu/phonotactic/about). These frequencies were matched across familiarization and test items. None of the items had phonological neighbors based on a search of the Washington University Speech and Hearing Laboratory’s Neighborhood Database (http://128.252.27.56/Neighborhood/NeighborHome.asp).

Richtsmeier et al. (2011) only observed phonotactic generalization in an experiment which combined multiple word types with talker variability. Following that finding, multiple talkers were employed here. Recordings of all familiarization and test items were obtained from seven adult female speakers of a Midwestern dialect of American English. The recordings were made in a sound booth following model productions made by the first author. This process was implemented to ensure that acoustic cues for medial consonants and the prosodic contours were produced faithfully. In particular, the talkers were encouraged to produce the stop at the end of the /pt/, /mk/, and /fp/ sequences with some aspiration. Recordings were later scrubbed of acoustic artifacts such as breaths and tongue clicks and scaled for intensity using Praat software (Boersma & Weenink, 2021). Productions from five of the talkers were used for the familiarization items; productions from the other two talkers were used for the test items. These same talkers produced the familiarization and test items in the prosody experiment.

#### Experimental frequency.

Participants were familiarized with the learning targets during a listening-only familiarization phase, and the experimental frequency of the targets varied as a within-subjects factor, with two targets in the low experimental frequency condition and two in the high experimental frequency condition. Children were familiarized with low experimental frequency targets in just one familiarization item (the items in italics in Table 2). Participants heard that item five times from a single talker, that is, from the same recording of just one of the five talkers who produced the familiarization items. High experimental frequency targets appeared in three familiarization items. Participants heard each item five times, each from a different talker, that is, a single recording from each of the five talkers who produced the familiarization items. Thus, high experimental frequency was a combination of high word-type frequency and talker variability (Richtsmeier, 2011; Richtsmeier et al., 2011). Several previous studies suggest that high experimental frequency can lead to greater production accuracy in children (Edeal & Gildersleeve-Neumann, 2011; Plante et al., 2011; Richtsmeier et al., 2009; Richtsmeier & Moore, 2020; Service et al., 2014), a finding that is consistent with the augmentative effect that high natural language frequency has on production accuracy (Beckman & Edwards, 2000; Edwards et al., 2004; Masdottir & Stokes, 2016; Munson, 2001; Storkel, 2015). The assignment of items to the two experimental frequencies was counterbalanced across four lists. Additionally, make-believe animals were assigned to different items in each list.

### Procedure

The procedures, including informed consent, were approved by the Internal Review Board at Purdue University. Children participated over 5 weeks, one visit per week. The first session included only standardized testing; participants completed a hearing screening, the SPELT-III language test, and the CMMT nonverbal skill test. Other standardized or normative data were collected following the experiments during the other four sessions. The first experiment was completed at the start of the second and third sessions, and the second experiment was completed at the start of the fourth and fifth sessions. Similar numbers of children completed the phonotactics experiment first (*n* = 16) and the phonotactics experiment second (*n* = 13). All sessions were held in a quiet room in a university building. Throughout each session, participants were seated in a Rifton chair with an attachable tabletop, approximately 10 feet from a monitor and speakers. Caregivers were seated nearby.

Before the start of the experiment, the experimenter explained that the child would hear the names of “funny, make-believe animals,” and that the child’s task during familiarization was to watch the animals and listen to their names. The first test block followed immediately, and the second test block was completed in the subsequent session 1 week later. At the start of each test block, participants were told that they would repeat the names of some new animals. Children heard and repeated each test item immediately. Productions of test items were therefore imitations, indicating that there was no requirement for children to learn form-referent pairings. Although children typically required one or two prompts for the first few productions of the first test block, they eventually learned the task and were able to proceed without prompts. Children had nine opportunities to produce each item during a test block. Cases where children did not produce an item were minimal (20 missing productions, or less than 1% of all attempts), and for all participants, there were always five or more productions of each word in each test block.

The experiment was controlled by Paradigm software (Paradigm, 2015) which presented familiarization items in random order. In contrast, test items were presented in a predetermined, pseudorandom order such that the same word was repeated no more than twice in a row.

### Analysis

Children’s productions were recorded digitally for transcription. The dependent measure of interest was production accuracy of the word-medial consonant sequence and was based on those transcriptions. Productions were transcribed by the first author and were converted to points based on a system adapted from Edwards et al. (2004). A correctly produced consonant was given a score of 3. A consonant that differed from the target by one feature (i.e., by voicing, place of articulation, or manner of articulation) was given a score of 2. Any other consonant or a sequence of two or more consonants (e.g., the [st] in [mæstpəm] produced for /mæfpəm/) was given a score of 1. If no consonant was heard, a score of 0 was given. Accuracy for the two consonants of a target sequence was then added, meaning that accuracy for each production ranged from 0 to 6. A second transcriber scored 424 word-medial sequences from 19 of the participants or approximately 20% of the data. Reliability between the two sets of transcriptions was 90.0% overall (82.3% for the first consonant; 97.6% for the second consonant). Lower accuracy for the first consonant is consistent with studies of similar experimental items (Richtsmeier & Moore, 2020). It likely reflects challenges related to producing and perceiving codas, as the first consonant of the sequence was the coda of the first syllable.

As described in the Introduction, a high-frequency advantage has been observed in other experiments. Assuming that statistical learning contributes to the language-wide high-frequency advantage, we would expect the experimental high-frequency advantage to persist over time. Our hypothesis was therefore that a main effect of experimental frequency would be observed, with greater accuracy for high experimental frequency sequences, and that this effect would persist or be enhanced across the two sessions. A null effect of experiment order was expected, but as an important step for counterbalancing, it was included in the statistical analyses. In sum, the three independent factors included were experimental frequency, session, and experiment order.

Accuracy scores for each production of a word-medial sequence were entered separately into linear mixed-effects models in R statistical software using the lmerTest package (Kuznetsova et al., 2020). We followed recommendations for mixed-model analyses described by Baayen et al. (2008). In particular, we began with a baseline model with main effects that was then compared with more specific models containing interactions.

For the baseline model, the main effects of experiment order, experimental frequency, and session were included. To evaluate the presence of a possible interaction between experimental frequency and experiment order, an alternative model allowed experimental frequency and experiment order to interact. The two models were then compared using a likelihood ratio test that was implemented with the anova function in R (Kuznetsova et al., 2017). If the alternative model had significantly lower deviance than the baseline model, it was considered the optimal model, and a significant interaction would be broken down into simple effects or separate analyses of each experiment order. Because all factors had just two levels, no contrast analyses were necessary beyond the analysis of simple effects. Alternatively, the baseline model would be considered optimal and interpreted for significant effects. Random effects for participant and item intercepts were included in both models. Random slopes were not included because models with them failed to converge.

Although mixed-effects models appear to be robust when analyzing a variety of data distributions (Schielzeth et al., 2020), to address concerns about the validity of the statistical approach, we completed three additional analyses: 1) production accuracy was coded as correct-incorrect, 2) production accuracy was coded as the arcsine transformation of the percentage of features produced correctly, and 3) the results were entered into a multinomial logistic regression. The results of these analyses were consistent with the mixed-effect model analysis reported below, and they are included in Appendix B. Analysis codes and data for the analyses, as well as data from the reliability analysis, are available at https://osf.io/ruf85/?view_only=fb021c494318415492be6e28853bd753.

## Results

**Figure 1.**
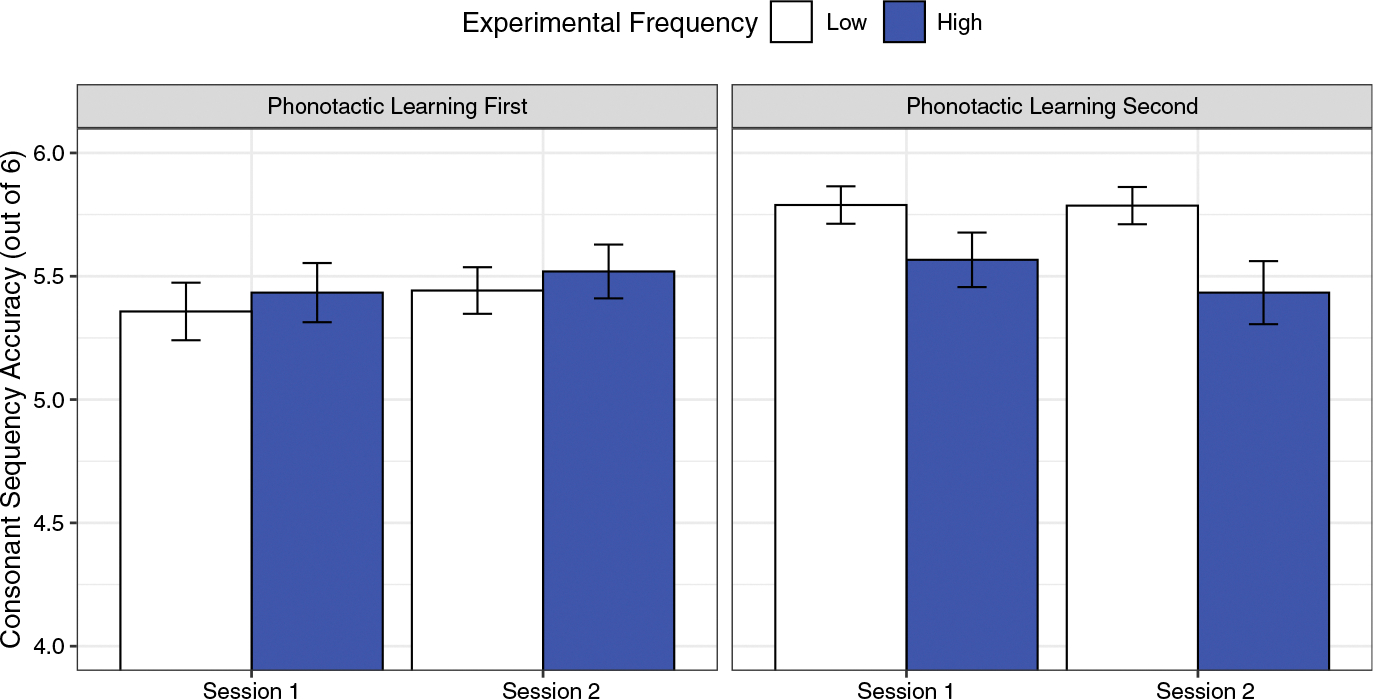
A comparison of the effects of experimental frequency and experiment order on consonant sequence accuracy. The bars reflect a limited range of accuracy values, from 4 to 6, to highlight differences between conditions. Error bars reflect standard errors.

Figure 1 presents the average accuracy in both the high and low experimental frequency conditions across the two experimental orders. In the figure, accuracy is collapsed across words and multiple productions. Mean accuracy across all conditions and participants was 5.53. Visual analysis of the figure suggests that participants were slightly more accurate in the high experimental frequency condition when the phonotactics experiment came first (High *M* = 5.47, *SD* = 0.91 vs. Low *M* = 5.40, *SD* = 0.94), but when the phonotactics experiment came second, they were more accurate in the low experimental frequency condition (High *M* = 5.50, *SD* = 0.87 vs. Low *M* = 5.79, *SD* = 0.52).

The baseline mixed-effects model included experiment order, experimental frequency, and session as main effects. Using a log-likelihood ratio test, the baseline was then compared with an alternative model in which experiment order and experimental frequency were allowed to interact. The results of the model comparison appear in Table 3 below. The alternative model had lower information criterion scores (AIC and BIC) and a lower log-likelihood value. Furthermore, it was significantly better when explaining the data (χ2 = 29.98, *df* = 1, *p* < .001).

As the alternative model was significantly better at explaining the data, it is summarized in Table 4 below. Contrary to our hypotheses, there was no significant effect of session (*β* = .01, *SE* = .03, *t* = 0.45, *p* = .653). Although there was a main effect of experimental frequency (*β* = .29, *SE* = .05, *t* = 5.79, *p* < .001), the experiment order × experimental frequency interaction was significant (*β* = −.37, *SE* = .07, *t* = −5.50, *p* < .001). To better understand that interaction, separate mixed-effects analyses were completed to examine experimental frequency in each experiment order condition.

Accuracy was marginally higher in the high experimental frequency condition for the phonotactic learning first data (*β* = −.08, *SE* = .05, *t* = −1.84, *p* = .066). This marginal high-frequency advantage is consistent with previous studies that report a benefit to high experimental frequency (e.g., Plante et al., 2011; Richtsmeier et al., 2009; Richtsmeier & Goffman, 2017; Richtsmeier & Good, 2018). In the high experimental frequency condition for the phonotactic learning second data, however, accuracy was significantly lower (*β* = .29, *SE* = .04, *t* = 6.51, *p* < .001). Thus, when participants completed the experiment second, a reversal of the high-frequency advantage was observed. Furthermore, the null effect of session indicates that these frequency effects were sustained across both sessions (see Figure 1).

As noted in the Participants section, there was an unplanned imbalance across the two experiment orders in terms of the participants’ sex, with a greater proportion of females in the phonotactic learning first condition. Sex was not expected to influence the results of the study, but because it was potentially confounded with experiment order, a new mixed-effects model was analyzed, similar to the alternative model above, with sex in place of experiment order as the predictive factor in the interaction with experimental frequency. This model with a sex × experimental frequency interaction was then compared with the alternative model from above. If sex differences are responsible for the different effects of experimental frequency, this model should provide a better fit than the alternative model from above. However, the alternative model with experiment order proved to be optimal (χ2 = 20.199, *df* = 1, *p* < .001). As such, it does not appear that the experiment order × experimental frequency interaction is the result of sex differences in sensitivity to experimental frequency.

## Discussion

In this study, children completed a statistical learning experiment that examined phonotactic learning either before or after a separate statistical learning experiment. For the phonotactic learning experiment reported here, the learning targets were word-medial consonant sequences. Children heard those sequences in either a single familiarization item produced by one talker (low experimental frequency) or in three familiarization items each produced by multiple talkers (high experimental frequency). Learning was probed by comparing the high and low experimental frequency conditions, and a difference in accuracy for high- and low-frequency targets was taken to signal learning. To assess learning over time, the experiment took place over two separate sessions a week apart. The key finding is that learning of the phonotactic sequences was influenced by the order in which participants completed the two experiments. When participants completed phonotactic learning first, there was a trend towards greater accuracy following high experimental frequency familiarization. In contrast, when participants completed phonotactic learning second, participants were significantly more accurate following the low experimental frequency familiarization. There was no main effect of session, suggesting that these learning effects were consistent over time. Therefore, the effect of experimental frequency, and by extension learning, primarily depended on the order in which the two experiments were completed. Below we describe a potential explanation for this surprising result. We also make recommendations for how this result could be replicated and extended. We argue that, as in the real world, experimental intermixing of statistical cues represents an important, albeit unanticipated, research direction—one that could reflect learning conditions experienced in the real world.

Before turning to the experiment order × experimental frequency interaction, the original motivation for the study should be considered, that is, whether experimental frequency effects persist over time. Although the null effect of session indicates that learning was sustained over time, a marginally significant effect was observed in the phonotactics learning first condition, providing relatively muted support for the idea that statistical learning underpins broader language-wide frequency effects in child speech accuracy. Two considerations may help for the interpretation of this result. First, as discussed by Richtsmeier and Goffman (2017), if we estimate learning effects over 1 week using normative speech development data (Smit, 1993), experimental frequency effects should appear to be quite small. In other words, on the scale of 1 week, typical speech development appears to involve very small changes in accuracy, smaller even than the high-frequency advantage observed in the phonotactic learning first condition. Second, speech accuracy appears to be differentially sensitive to perceptual learning and production practice, with perceptual learning of the kind examined here having relatively small effects compared to production practice (Richtsmeier & Moore, 2020; Service et al., 2014). In this study, children engaged in significant production practice or nine productions of each test item in each session. Speech motor practice may solidify representations and thus result in reduced flexibility over the course of learning (Richtsmeier & Goffman, 2017). Thus, statistical learning likely does contribute to language-wide frequency effects in child speech accuracy, but the impact may be small, and it is important to consider *what* is frequent, for example, whether it is a frequent input or frequent production practice.

We turn now to the experiment order × experimental frequency interaction. There is a substantial body of literature showing that frequent consonants and phonotactic sequences tend to be acquired earlier and produced more accurately (Edwards et al., 2015). To our knowledge, there is no literature indicating that high frequency should sometimes result in lower speech accuracy, although frequency effects in word learning are somewhat less straightforward (Heisler & Goffman, 2016; Hoover et al., 2010). The present findings are surprising in that, when considering raw accuracy, we see what we refer to as a high-frequency disadvantage, or, conversely, a low-frequency advantage. It is currently unclear which of these possible explanations is best. An interpretation of the results as a “high-frequency disadvantage” is appealing because it is difficult to formulate an explanation for the results without referring to high frequency and because this choice is consistent with principles of non-associative learning. In other words, whether learning results in more or less of a specific behavior like correct production, that learning is proportional to the frequency of the stimulus (Ioannou & Anastassiou-Hadjicharalambous, 2018).

However, it is also possible to interpret the results as a “low-frequency advantage.” This interpretation follows from the enhanced levels of production accuracy observed for low experimental frequency sequences when phonotactic learning followed prosodic learning (see Figure 1). It is also consistent with a “less is more” or capacity limitation hypothesis. It may be that focusing young learners on the higher prosodic levels of the phonological hierarchy reduced their capacity to later process multiple phonotactic exemplars. That is, when the child initially attended to the prosodic statistical learning task, lower frequency input resulted in enhanced learning in the phonotactic learning task that followed (e.g., Conway et al., 2003; Elman, 1993). For the remainder of the discussion, we will use the term “low-frequency advantage,” but future research is certainly needed to determine whether high frequency or low frequency was the driving force in the phonotactic learning second condition.

Because a low-frequency advantage is essentially the opposite of what is expected, it is important to consider whether the result is spurious, that is, a case of Type I error. One way to manage Type 1 error is to impose a more stringent significance level, or α, as is often done when multiple statistical tests are run. No adjustment was made to α in this study, primarily because a single omnibus statistical test was run. However, even if we reduced α to 0.001, the low-frequency advantage, reported by R software as *p* = 1.21 × 10^−10^, would remain significant.

A second possible explanation is that the result reflects inherent, confounded differences between the two participant groups. Sex is one possible confound, but when we conducted an analysis with sex replacing experiment order, the overall model was inferior to the model which included experiment order. Furthermore, as can be seen in Table 1, the two participant groups were relatively well matched across a number of developmental measures including age, articulatory development, nonverbal cognitive skill, expressive vocabulary, receptive vocabulary, expressive syntax, and verbal working memory. Unintended differences in between-subjects designs are always possible, but our post hoc analyses in search of such confounds do not appear to nullify the result.

A third possibility is that boredom, fatigue, forgetting, attenuation of learning, or some time-related confound is behind the unexpected result. Time-related confounds do limit the interpretation of these data. However, boredom, fatigue, and attenuation all imply that learning should be reduced over time. In this case, if we estimate learning as the difference between high and low experimental frequency conditions divided by their pooled standard deviations (Cohen, 1988), there was a larger learning effect for participants who completed the experiment second compared to those who completed the experiment first (0.31 vs. 0.11). Thus, the results indicate a need for additional explanations.

Related to the concerns above, interpretation of the results is challenging because of the limited set of materials used. It is unknown whether the low-frequency advantage would be observed with a different set of items or when probing learning of a different phonological generalization, although this limitation is typical of psycholinguistic research. We do note that a low-frequency advantage was not observed in the analyses of the prosody learning experiment, although no effect of experimental frequency of any kind was observed in those data. Without an experimental frequency effect, the prosody learning data do not allow either confirmation or rejection of a similar effect and therefore do not aid in the interpretation of the present findings.

Setting aside the possibility that the low-frequency advantage is the result of a confound or an artifact of the experimental design, another explanation is that competing statistical patterns may influence learning. Although a low-frequency advantage may have been unanticipated, there is a body of literature in which typical statistical learning effects have not been observed, sometimes referred to as multistream statistical learning. Learners in multistream statistical learning experiments are presented with two incompatible or competing inputs. In general, this literature suggests that it is difficult for learners to simultaneously retain knowledge of both inputs. For example, Weiss et al. (2009) report four statistical word segmentation experiments exploring how adults interpret and learn from two inputs presented in sequence. In their statistical word segmentation task, participants heard a continuous stream of syllables like *b*ə*tigusɪt*ʃə*viv*ʊ*bosætog*ʊ*t*ʃ*a*. Some syllables always occurred in a sequence, such as *b*ə, *ti*, and then *gu*, meaning that *b*ə*tigu* functioned like a word in the stream. However, the authors interleaved two inputs in the stream such that *b*ə*tigu* functioned as a word during some sections of the stream but not others. The participants were tested on their ability to discriminate words from the first input like *b*ə*tigu* from part-words like *tigusɪ*. Adults learned the patterns from both inputs when each input was spoken by a different talker, but not when the same talker produced both inputs. Weiss and colleagues suggest that learning fails when participants conflate the statistics of the two inputs, as may be expected when all stimuli come from a single talker. Similar failures to learn from a competing input have been reported by Benitez et al. (2020), Gebhart et al. (2009), and Potter and Lew-Williams (2019).

To explain the low-frequency advantage, it may be that the participants in the phonotactic learning second condition were conflating aspects of the prosodic and phonotactic familiarizations or that the focus on prosodic learning, which occurs at a higher level of the phonological hierarchy, interfered with phonotactic learning. One concern with connecting our experiment with the multistream statistical learning literature is that our two experiments had relatively little phonological overlap compared to the overlap created by Weiss et al. (2009) and others. In our experiments, participants heard words one at a time, rather than hearing a syllable stream. Furthermore, the two experiments exposed participants to phonotactic and prosodic patterns, respectively, whereas most multistream statistical learning studies simply change the statistics of which syllables go together to form words. Thus, one could argue that there was not sufficient phonological overlap in our two experiments to motivate any sort of interaction. However, an alternative possibility is that it was the very inclusion of two different levels of the phonological hierarchy, one prosodic and one phonotactic, that led to our unanticipated results. Certainly, the interaction was unexpected and unplanned, but there are hints as to why an interaction was possible here.

First, there was a significant difference in the direction of experimental frequency effects that is not easily dismissed as Type I error, did not appear to depend on measured characteristics of the participants, and resisted a simple time-based explanation like fatigue or boredom.

Second, the familiarization talkers in the two experiments overlapped. Weiss et al. (2009) found that adult learners struggle to distinguish syllable streams with different statistics if they are produced by the same talker. Perhaps children in our study were encouraged to conflate the two experiments because they heard the same talkers during the familiarizations.

Third, although our study is unique in finding a low-frequency advantage, it is possible that studies by Weiss et al. and others include a similarly unexpected effect implicitly, but it simply was not measured. In other words, the multistream statistical learning literature reports statistical tests that either confirm learning or do not, and it is not possible to say whether unexpected learning occurred. In our study, we compared speech accuracy across high and low experimental frequency conditions. Compared to the multistream statistical learning literature, this relative difference between these conditions is unique in being able to tap into the directionality of the learning effect. This design is therefore potentially advantageous for future research that looks at how learning in one experiment or familiarization condition interacts with another experiment or familiarization condition.

Fourth, there is some evidence that participants in statistical learning tasks can make unexpected or surprising generalizations. In an infant statistical learning study by Gerken and Quam (2017), 11-month-olds were exposed to just one input. Infants heard novel CVCV words containing a target phonological pattern, the pattern being either shared place of articulation (*poba* contains two labials) or shared voicing (*dova* contains two voiced consonants). Although only one pattern was present in the exposure words, some infants heard the words in an order that allowed for a local phonological generalization, for example, when two or three adjacent words started with the same consonant. When local generalizations were present, infants did not appear to learn the more global phonological patterns for place of articulation or voicing. When those local generalizations were removed, however, infants learned the more general patterns. Although Gerken and Quam did not initially expect the local generalizations, the finding ultimately opened up new considerations about the time course and sensitivity of infants to input statistics.

Such an unexpected generalization is possible in our study. For example, participants in the prosody experiment were exposed to items like /do.sə/ and /po.fə/ with the same strong-weak stress pattern present in all of the phonotactic items. Perhaps participants in the prosodic learning experiment learned to expect that the strong-weak stress pattern should only be present in CV.CV syllables lacking consonant sequences, or lacking closed syllables, thereby making the CCs in the CVC.CVC items from the phonotactic learning experiment, especially the frequent CCs, unexpected. Other explanations likely exist and are worth considering. Here, we simply note that there is some precedent for both interactions of statistical learning inputs as well as surprising generalizations made by learners.

In sum, there is enough evidence in the literature of interacting or surprising statistical learning effects that the experimental frequency × experiment order interaction reported here should be followed up on. Certainly, additional research is necessary. A key next step will be to verify that participants attempt to impose patterns from the first input onto the second. Here and in other multistream statistical learning studies, the authors have verified that participants did not exhibit an expected pattern, but they have not specifically probed for the unintended generalization. In other words, what is needed is explicit evidence that participants are applying a pattern from the first experiment to the second experiment. Such a study is warranted, particularly in the area of child speech development. Statistical learning researchers typically test a statistical learning pattern and infer whether this pattern was learned or not. In the real world, linguistic patterns are intermixed, yet they must be learnable for children to arrive at an adult state. The present findings suggest that learners may struggle, at least initially, when faced with multiple patterns. However, it also opens up novel questions regarding when multiple patterns are difficult to learn from and how learners overcome those difficulties.

## Conclusion

In a study of the learning of phonotactic sequences, we reported an expected but marginal high-frequency advantage and an unexpected but significant low-frequency advantage. Although the cause of the latter effect remains unclear, there is preliminary evidence that the presence of another learning experiment that occurred prior to the phonotactic experiment may be responsible for the unexpected effect. To the extent that learners conflated learning across the two experiments, future research should explore when such conflations or interactions occur.

## Supplementary Material

Appendix A

Appendix B

**Supplementary material.** To view supplementary material for this article, please visit https://doi.org/10.1017/S0142716423000449

## Figures and Tables

**Table 1.** Averages and standard deviations for age in months, standardized test scores, a nonword repetition task, and average production accuracy for each experiment. A statistical comparison of the two experiment order groups appears in the rightmost column

	*M* (*SD*)		*t* statistic (*p* value)
	Phonotactics First	Phonotactics Second	
Female/Male	11/5	6/7	4.74 (*p* = 0.03)*
Mean age in months^a^	56.4 (52–67)	58.8 (45–69)	0.96 (*p* = 0.32)
BBTOP standard scores	99.77 (12.47)	102.88 (9.12)	−0.75 (*p* = 0.46)
CMMS standard scores	116.15 (11.51)	111.31 (9.41)	1.2 (*p* = 0.24)
EVT standard scores	109.62 (7.29)	110.88 (9.13)	−0.39 (*p* = 0.70)
PPVT-4 standard scores	119.85 (11.65)	114.31 (9.65)	1.35 (*p* = 0.19)
SPELT-III standard scores	113.85 (7.38)	114.56 (8.22)	−0.24 (*p* = 0.82)
Nonword repetition percent phonemes correct	78.68 (7.76)	74.65 (12.47)	0.94 (*p* = 0.36)
Average accuracy: Phonotactics^b^	5.43 (0.43)	5.64 (0.25)	1.49 (*p* = 0.15)

*Note.* BBTOP = Bankson-Bernthal Test of Phonology, CMMS = Columbia Mental Maturity Scale, EVT = Expressive Vocabulary Test-1, PPVT-4 = Peabody Picture Vocabulary Test-4, SPELT-III = Structured Photographic Expressive Language Test-3.

a The number in parentheses for ages in months is the range of ages rather than the standard deviation.

b Accuracy in the phonotactics experiment is on a scale from 0 to 6.

* Counts of females and males were compared using a *χ*^2^ test. The test was significant and will be covered in the Results and General Discussion.

**Table 2.** The learning targets, familiarization items, and test items for the phonotactic learning experiment. Syllable boundaries are indicated with a period. In the high experimental frequency condition, participants heard all three familiarization items involving a target sequence. In the low experimental frequency condition, participants only heard the italicized familiarization item

	Target	Familiarization items			Test items
Phonotactics Experiment	/pt/	dap.tən	zeɪp.təs	*sεp.təf*	bip.təm
	/zm/	koz.mət	lɪz.məs	*taɪz.mək*	pεz.mef
	/mk/	gum.kəf	tæm.kən	*dɪm.kəs*	fom.kəp
	/fp/	nɪf.pən	ʃeɪf.pək	*kof.pət*	mæf.pəm

**Table 3.** The results of the model comparison for the phonotactics experiment. The baseline model with main effects was compared to an alternative model in which experiment order and experimental frequency were allowed to interact

Model	df	AIC	BIC	Deviance	*χ* ^2^	df	*p*
Baseline model	7	4,769.2	4,808.6	4,755.2	22.37	1	<.001
Alternative model	8	4,748.8	4,793.9	4,732.8			

**Table 4.** Summary of the alternative mixed-effects model of the phonotactics experiment. Statistically significant fixed effects are shown in bold. The number of observations was 2068

Fixed effects		*β*	SE	df	*t*	*p* (>|*t*|)
**Intercept**		**5.51**	**0.14**	**22.00**	**38.16**	**<.001**
Experiment order		−0.05	0.14	31.99	−0.37	.711
**Experimental frequency**		**0.23**	**0.05**	**2038.57**	**4.68**	**<.001**
Session		0.01	0.03	2035.96	0.45	.653
**Experiment order × Experimental frequency**		**−0.31**	**0.07**	**2037.54**	**−4.74**	**<.001**
Random Effects	Variance	Standard Deviation				
Participant (intercept)	0.13	0.36				
Item (intercept)	0.03	0.17				
