# Supplementary material for: Statistical learning of phonotactics by children can be affected by another statistical learning task
Source: Appl Psycholinguist. Author manuscript; Available in PMC 2026 May 1. (PMC13132535; doi:10.1017/s0142716423000449)
Supplement: Appendix A [file NIHMS2159757-supplement-Appendix_A.docx]

**Appendix A**

Table A1. The learning targets, familiarization items, and test items for the prosodic learning experiment. Results of this experiment will be reported elsewhere. Syllable boundaries are indicated with a period. In the high experimental frequency condition, participants heard all three familiarization items. In the low experimental frequency condition, participants only heard the italicized familiarization item.

|  | Target | Familiarization Items | | | Test Items |
| --- | --- | --- | --- | --- | --- |
| Prosodic Learning  Experiment | 2-syllable SW | re.də | ti.də | *do.sə* | po.fə |
|  | 4-syllable SW | do.lə.re.sə | so.lə.ti.rə | *la.tə.so.rə* | mi.fə.po.bə |
|  | 2-syllable WS | lə.do | tə.re | *sə.la* | bə.mi |
|  | 4-syllable WS | də.ti.rə.la | rə.so.tə.do | *tə.la.sə.re* | pə.fa.mə.be |
