# Supplementary material for: Statistical learning of phonotactics by children can be affected by another statistical learning task
Source: Appl Psycholinguist. Author manuscript; Available in PMC 2026 May 1. (PMC13132535; doi:10.1017/s0142716423000449)
Supplement: Appendix B [file NIHMS2159757-supplement-Appendix_B.docx]

**Appendix B**

This appendix contains three alternative analyses to supplement the mixed-effect model analysis reported in the manuscript. Two of the analyses (Correct/Incorrect and Arcsine Transformation) involve transformations of the dependent variable. A third analysis (Multinomial) uses an alternative statistical test, namely, a multinomial logistic regression. The important take-away from all three analyses is that, for their respective alternative models, they all find a significant interaction of experiment order and experimental frequency. Furthermore, the alternative models are all significantly better at describing the data compared to a baseline model without the experiment order × experimental frequency interaction.

Correct/Incorrect Analysis

Table B1 – The results of the model comparison for the correct/incorrect (C/I) coding of children’s production accuracy. The baseline model with main effects was compared to an alternative model in which experiment order and experimental frequency were allowed to interact.

| Model | df | AIC | BIC | Deviance | χ^2^ | df | *p* |
| --- | --- | --- | --- | --- | --- | --- | --- |
| Baseline C/I model | 7 | 2595.1 | 2634.5 | 2581.1 | 20.88 | 1 | <.001 |
| Alternative C/I model | 8 | 2576.2 | 2621.3 | 2560.2 |  |  |  |

As the alternative model was significantly better at explaining the data, it is summarized in Table B3 below. There was no significant effect of session (*β* = .002, *SE* = .02, *t* = 0.12, *p* = .908). Although there was a main effect of experimental frequency (*β* = .08, *SE* = .03, *t* = 2.60, *p* = .01), the experiment order × experimental frequency interaction was significant (*β* = -.18, *SE* = .04, *t* = -4.58, *p* < .001).

Table B3 – Summary of the alternative mixed effects model for the correct/incorrect (C/I) coding of children’s production accuracy. Statistically significant fixed effects are shown in bold. The number of observations was 2068.

| Fixed Effects | *β* | SE | df | *t* | *p* (>\|*t*\|) |
| --- | --- | --- | --- | --- | --- |
| **Intercept** | **1.71** | **0.10** | **15.1** | **17.42** | **<.001** |
| Experiment Order | -0.05 | 0.09 | 31.46 | -0.62 | .541 |
| **Experimental Frequency** | **0.08** | **0.03** | **2038** | **2.60** | **.01** |
| Session | 0.002 | 0.02 | 2036 | 0.12 | .908 |
| **Experiment Order × Experimental Frequency** | **-0.18** | **0.04** | **2037** | **-4.52** | **<.001** |
| Random Effects | Variance | Standard Deviation |  |  |  |
| Participant (intercept) | 0.05 | 0.22 |  |  |  |
| Item (intercept) | 0.02 | 0.14 |  |  |  |

Arcsine Transformation Analysis

Table B4 – The results of the model comparison for the arcsine transformation (AT) coding of children’s production accuracy. The baseline model with main effects was compared to an alternative model in which experiment order and experimental frequency were allowed to interact.

| Model | df | AIC | BIC | Deviance | χ^2^ | df | *p* |
| --- | --- | --- | --- | --- | --- | --- | --- |
| Baseline AT model | 7 | 1012.2 | 1051.6 | 998.2 | 24.53 | 1 | <.001 |
| Alternative AT model | 8 | 989.67 | 1034.7 | 973.67 |  |  |  |

As the alternative model was significantly better at explaining the data, it is summarized in Table B5 below. There was no significant effect of session (*β* = .002, *SE* = .06, *t* = 0.12, *p* = .903). Although there was a main effect of experimental frequency (*β* = .08, *SE* = .02, *t* = 4.25, *p* < .001), the experiment order × experimental frequency interaction was significant (*β* = -.13, *SE* = .03, *t* = -4.97, *p* < .001).

Table B5 – Summary of the alternative mixed effects model for the correct/incorrect (C/I) coding of children’s production accuracy. Statistically significant fixed effects are shown in bold. The number of observations was 2068.

| Fixed Effects | *β* | SE | df | *t* | *p* (>\|*t*\|) |
| --- | --- | --- | --- | --- | --- |
| **Intercept** | **1.36** | **0.06** | **19.42** | **22.02** | **<.001** |
| Experiment Order | -0.02 | 0.06 | 31.69 | -0.32 | 0.749 |
| **Experimental Frequency** | **0.08** | **0.02** | **2038** | **4.25** | **<.001** |
| Session | 0.002 | 0.01 | 2036 | 0.12 | .903 |
| **Experiment Order × Experimental Frequency** | **-0.13** | **0.03** | **2037** | **-4.97** | **<.001** |
| Random Effects | Variance | Standard Deviation |  |  |  |
| Participant (intercept) | 0.02 | 0.15 |  |  |  |
| Item (intercept) | 0.006 | 0.08 |  |  |  |

Multinomial Analysis

Table B6 – The results of the model comparison for the multinomial logistic regression (MLR) analysis of children’s production accuracy. Note that this analysis includes the same dependent variable as the main mixed-effects model analysis presented in the manuscript. A baseline multinomial model with main effects was compared to an alternative multinomial model in which experiment order and experimental frequency were allowed to interact.

| Model | Parameters | AIC | Log Likelihood | LR.stat  (χ^2^) | df | *p* |
| --- | --- | --- | --- | --- | --- | --- |
| Baseline MLR model | 9 | 3255.5 | -1618.8 | 36.478 | 1 | <.001 |
| Alternative MLR model | 10 | 3221.0 | -1600.5 |  |  |  |

As the alternative model was significantly better at explaining the data, it is summarized in Table B7 below. There was no significant effect of session (*β* = .03, *SE* = .10, *z* = 0.32, *p* = .746). Although there was a main effect of experimental frequency (*β* = .99, *SE* = .18, *z* = 5.38, *p* < .001), the experiment order × experimental frequency interaction was significant (*β* = -1.37, *SE* = .23, *z* = -5.93, *p* < .001).

Table B7 – Summary of the alternative multinomial logistic regression (MLR) analysis of children’s production accuracy. Statistically significant fixed effects are shown in bold. The number of observations was 2068.

| Fixed Effects | | *β* | | SE | | *z* | | *p* (>\|z\|) | |  |
| --- | --- | --- | --- | --- | --- | --- | --- | --- | --- | --- |
| Experiment Order | | -0.05 | | 0.51 | | -0.09 | | 0.929 | |  |
| **Experimental Frequency** | | **0.99** | | **0.18** | | **5.38** | | **<.001** | |  |
| Session | | 0.03 | | 0.10 | | 0.32 | | .746 | |  |
| **Experiment Order × Experimental Frequency** | | **-1.37** | | **0.23** | | **-5.93** | | **<.001** | |  |
| Random Effects | Variance | | Standard Deviation | |  | |  | |  | |
| Participant (intercept) | 1.68 | | 1.30 | |  | |  | |  | |
| Item (intercept) | 0.44 | | 0.66 | |  | |  | |  | |
